# Anti-*Toxoplasma gondii* screening of MMV pandemic response box and evaluation of RWJ-67657 efficacy in chronically infected mice

**DOI:** 10.1017/S0031182023000999

**Published:** 2023-11

**Authors:** Bruna Ramos dos Santos, Amanda Bruno da Silva Bellini Ramos, Renata Priscila Barros de Menezes, Marcus Tullius Scotti, Fábio Antônio Colombo, Marcos José Marques, Juliana Quero Reimão

**Affiliations:** 1 Departamento de Morfologia e Patologia Básica, Faculdade de Medicina de Jundiaí, Laboratory of Preclinical Assays and Research of Alternative Sources of Innovative Therapy for Toxoplasmosis and Other Sicknesses (PARASITTOS), Jundiaí, Brazil; 2 Departamento de Análises Clínicas e Toxicológicas, Faculdade de Ciências Farmacêuticas, Universidade Federal de Alfenas, Alfenas, Brazil; 3 Programa de Pós-graduação em Produtos Naturais e Sintéticos Bioativos (PgPNSB), Instituto de Pesquisa em Fármacos e Medicamentos (IPeFarM), Universidade Federal da Paraíba, Campus I, Cidade Universitária, João Pessoa, Brazil

**Keywords:** antitoxoplasmic compound, bioinformatics, drug discovery, drug repurposing, mice, pandemic response box, RWJ-67657, screening, *Toxoplasma gondii*, toxoplasmosis

## Abstract

Toxoplasmosis is a significant public health concern with limited therapeutic options. The medicines for malaria venture (MMV) developed the pandemic response box (PRB) containing 400 drug-like molecules with broad pathogen activity. The aim of this work is to evaluate PRB compounds for their anti-*Toxoplasma gondii* activity and identify promising candidates for further evaluation. Screening identified 42 selective compounds with half effective concentration (EC_50_) ranging from 2.4 to 913.1 nm and half cytotoxic concentration (CC_50_) ranging from 6 μm to >50 μm. Selectivity index (SI) values (CC_50_/EC_50_) ranged from 11 to 17 708. Based on its *in silico* and *in vitro* profile and its commercial availability, RWJ-67657 was selected for further studies. Molecular docking analysis showed RWJ-67657 is predicted to bind to *T. gondii* p38 mitogen-activated protein kinase (TgMAPK). Oral administration of RWJ-67657 (20 mg kg day^−1^/10 days) significantly reduced parasite burden in chronically infected mice compared to mock-treated group (*P* < 0.01). These findings highlight the PRB as a promising source for anti-*T. gondii* compounds, with several showing favourable drug properties, including MMV1634492, MMV002731, MMV1634491, MMV1581551, MMV011565, MMV1581558, MMV1578577, MMV233495 and MMV1580482, firstly described here as anti-*T. gondii* agents. RWJ-67657 emerges as a valuable drug candidate for experimental chronic cerebral toxoplasmosis therapy.

## Graphical abstract

**Figure:**
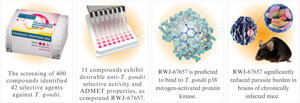

## Introduction

*Toxoplasma gondii* is an obligate intracellular protozoan parasite with high prevalence in a wide range of warm-blooded hosts (Antczak *et al*., 2016). With approximately 35% of the world's population affected during their lifetime, toxoplasmosis stands as the most prevalent parasitic zoonotic disease globally. In humans, toxoplasmosis can lead to severe complications, particularly in fetuses, new-borns and immunocompromised individuals (Akbari *et al*., 2022).

Despite its widespread impact, current treatment options for toxoplasmosis, including pyrimethamine and sulphadiazine, are outdated, limited in efficacy and often accompanied by toxic side effects. These drugs primarily target the acute phase of the infection and demonstrate restricted effectiveness against *T. gondii* tissue cysts, making them ineffective in the treatment of chronic infections. Additionally, the emergence of drug-resistant strains poses a significant concern (Da Silva *et al*., 2021). Consequently, there is a pressing need to develop novel therapeutics to address the limited availability of effective treatment options for this condition.

Mitogen-activated protein kinases (MAPKs), which are highly conserved across eukaryotes and essential for regulating intricate cellular processes in *T. gondii*, including proliferation, stress response and stage differentiation, represent promising candidates for drug development. Previous studies have demonstrated the inhibitory effects of human p38 MAPK inhibitors on *T. gondii* replication (Wei *et al*., 2002) and their efficacy in treating acute toxoplasmosis in mice (Wei *et al*., 2007).

Moreover, previous researches have showcased the potential of compounds provided by the ‘Medicines for Malaria Venture’ (MMV) organization for repositioning in the context of toxoplasmosis (Boyom *et al*., 2014; Spalenka *et al*., 2018; Cajazeiro *et al*., 2022; Dos Santos *et al*., 2023). In this study, the aim was to identify potential compounds from the MMV Pandemic Response Box (PRB) and explore their repositioning against toxoplasmosis using *in silico*, *in vitro* and *in vivo* approaches. The PRB is a collection of compounds meticulously selected from public domain databases based on clinical relevance and drug discovery potential. The composition includes 201 antibacterial, 153 antiviral and 46 antifungal compounds, showcasing a diverse molecular target range. Further details, including compound origins and target diseases, is available in Samby *et al*., 2022. Among the compounds investigated, the human p38 MAPK inhibitor RWJ-67657 was selected for further evaluation as a potential candidate to reduce parasite burden in the brains of chronically infected mice.

## Materials and methods

### Ethical standards

Experiments were performed using female C57BL/6 mice (4 weeks old) obtained from the animal breeding facility at the Universidade Federal de Alfenas, Brazil. All animals were handled in strict accordance with good animal practice as outlined in the Brazilian Guidelines for Care and Utilization of Animals from the Conselho Nacional de Controle e Experimentação Animal (CONCEA). The study was approved by the Ethics Committee for Animal Experimentation (Protocol 033/2021) of the Universidade Federal de Alfenas, and all efforts were made to minimize suffering.

### Cell and parasites

Human foreskin fibroblasts (HFF) were cultured in 25 cm^2^ culture flasks in Dulbecco's modified eagle's medium (DMEM) supplemented with 10% fetal bovine serum (FBS), L-glutamine (2 mm) and gentamicin (10 μg mL^−1^) (D10 medium) (Wang and Sibley, 2020) at 37°C under a 5% CO_2_ atmosphere. *Toxoplasma gondii* tachyzoites of the RH strain encoding a transgenic copy of *β*-galactosidase (type I, clone 2F1) (Castro *et al*., 2013) were maintained in HFF cells cultured in DMEM medium supplemented with 2% FBS, L-glutamine (2 mm), and gentamicin (10 μg mL^−1^) (D2 medium) at 37°C and 5% CO_2_. *Toxoplasma gondii* cysts of the ME49 strain were obtained from the brain tissue of animals chronically infected with the parasite, following a previously described method (Wang and Sibley, 2020).

### In vitro cytotoxicity

The concentration of 1 μm was employed to conduct the initial screening of all MMV PRB compounds against the host cell, allowing the identification of the least cytotoxic compounds at a concentration equivalent to that used in the screening against *T. gondii*, as previously described (Dos Santos *et al*., 2023). Subsequently, the determination of Half Cytotoxic Concentration (CC_50_) was performed using a 2-fold dilution series, ranging from 50 μm to 0.39 μm, for compounds that demonstrated up to 50% inhibition of HFF growth at 1 μm concentration. For both assays, HFF cells were seeded at a density of 5 × 10^4^ cells well^−1^ (in 100 μL volume) in 96-well microplates and allowed to adhere overnight. Subsequently, the cells were incubated with the test compounds for a duration of 72 h at 37°C in a 5% CO_2_ humidified incubator. Cell viability was assessed using the MTT assay, as described previously (Reimão *et al*., 2016). Following the 72-h incubation period, the culture medium in each well was replaced with 100 μL of phosphate-buffered saline (PBS), and 20 μL of MTT solution (5 mg mL^−1^) was added to each well. The plates were then incubated at 37°C for 4 h. Formazan extraction was performed by adding 80 μL of 10% SDS. After 18 h incubation in the dark at room temperature, the optical density of the formazan solution was measured at 550 nm using a microplate reader (Thermo Scientific Varioskan LUX). HFF cells incubated in the absence of drug treatment (DMSO control) were used as a viability control and pyrimethamine (PYR) was used as a reference drug (positive control). The percentage of DMSO in the control wells was maintained equivalent to that used in the highest compound dose tested in the assay. The viability of cells was expressed as a percentage relative to the optical density of untreated HFF cells, after normalization. The Selectivity Index (SI) was calculated as the ratio of CC_50_ against HFF cells to EC_50_ against *T. gondii* tachyzoites. The data presented in this study are representative of results obtained from 2 or more biological replicates. Dose-response inhibition curves (Log (inhibitor) *vs* normalized response – variable slope) were generated using Skanlt Software (Thermo Scientific).

### In vitro activity against *T. gondii*

An initial anti-*T. gondii* screening of all MMV PRB compounds was conducted at a concentration of 1 μm to identify the most active compounds at the lowest concentration (Spalenka *et al*., 2018). Subsequently, the Half Effective Concentration (EC_50_) was determined using a 2-fold dilution series, ranging from 2 μm to 15.62 nm, for all compounds that exhibited at least 80% inhibition of *T. gondii* growth at a concentration of 1 μm, including pyrimethamine, except for compounds MMV1580173 and MMV1580844, which were tested in the range of 8 to 0.062 nm. For both assays, 5 × 10^3^ HFF cells/well were seeded in 100 μL volume in 96-well plates and incubated overnight to allow for cell adherence. The wells were then emptied and refilled with fresh D2 medium containing 5 × 10^3^ RH-2F1 parasites in 100 μL volume, followed by incubation for 3 h at 37°C with 5% CO_2_. Subsequently, the infected cells were treated with the test compounds and incubated for 72 h at 37°C with 5% CO_2_. The evaluation of *β*-galactosidase activity was performed as previously described (Chin *et al*., 2007). Briefly, the parasites were incubated with 100 μL of lysis buffer (100 mm HEPES, 1 mm MgSO_4_, 0.1% Triton X-100, 5 mm DTT) for 15 min. The lysates were then mixed with 160 μL of assay buffer (100 mm phosphate buffer pH 7.3, 102 mm *β*-mercaptoethanol, 9 mm MgCl_2_) and 40 μL of 6.25 mm CPRG. Following a 30-minute incubation, the *β*-galactosidase activity was measured at 570 nm using a microplate reader (Thermo Scientific Varioskan LUX). HFF cells incubated in the absence of drug treatment (DMSO control) were used as a viability control, and PYR was employed as a reference drug (positive control). The viability of parasites was expressed as a percentage relative to the optical density of untreated parasite cells, after normalization. The data presented in this study are representative of results obtained from 2 or more biological replicates. Dose–response inhibition curves (Log (inhibitor) *vs* normalized response – variable slope) were generated using Skanlt Software (Thermo Scientific) and GraphPad Prism 8 software.

### Threshold criteria for selecting compounds

The present study employed the threshold criteria for selecting compounds that induce at least 80% inhibition of *T. gondii* at 1 μm and less than 50% inhibition of HFF at the same concentration, as previously described (Dos Santos *et al*., 2023). However, it's important to note that the compounds tested were specific to the respective libraries they belonged to. The earlier study by Dos Santos *et al*. (2023) focused on compounds from the MMV COVID box, whereas the present study assessed compounds from the MMV PRB. While the selection criteria were consistent, the compounds themselves were distinct due to the different libraries being investigated.

### In silico studies

To predict the absorption, distribution, metabolism, excretion and toxicity (ADMET) parameters of the compounds, the Swiss ADME open-access web tool was employed (Daina *et al*., 2017) (http://www.swissadme.ch). To predict the toxicity of the compounds, the OSIRIS Property Explorer (Sander *et al*., 2009) was used, considering parameters such as mutagenicity, tumorigenicity, reproductive effects and irritability. For absorption evaluation, factors such as membrane permeability, intestinal absorption and the substrate or inhibitor of glycoprotein P (P-gp) were considered. Specifically, compounds that did not violate more than 2 of the Lipinski rule criteria and had a logP consensus value not exceeding 4.15 were selected. Additionally, the compounds were assessed to ensure they were not substrates for the P-gp enzyme, which affects permeability. Distribution was evaluated based on factors including blood–brain barrier penetration (BBB).

The hybrid model for the TgMAPK protein was constructed using YASARA software (YASARA Biosciences GmbH, Vienna, Austria), based on the FASTA sequence of *T. gondii* MAPK family (ERK) MAPK-1 (EPR61015.1) retrieved from the UniProt database (https://www.uniprot.org/) (Brumlik *et al*., 2004; Krieger and Vriend, 2014). The stereochemical quality of the models was assessed using PROCHECK (Laskowski *et al*., 1993), which evaluated various stereochemical parameters such as torsional angles of the main chain, torsional angles of the side chain, steric clashes and planarity (Herrera-acevedo *et al*., 2021). A Ramachandran plot was generated in the same software to determine allowed and disallowed regions of the main amino acid chain. The structural quality was further evaluated using VERIFY 3D software (https://services.mbi.ucla.edu/SAVES/) to analyse the compatibility of the protein sequence with its 3D structure, and WHAT IF (https://swift.cmbi.ru.nl/servers/html/index.html) to assess various structural parameters including atomic contacts between residues. Quality *Z*-scores of dihedrals were calculated to determine the model's quality relative to high-resolution X-ray structures (Laskowski *et al*., 1993). Higher values indicate better quality, while negative values suggest that the homology model is less favourable compared to high-resolution X-ray structures. Molecular docking was performed using Molegro Virtual Docker version 6.0.1 (MVD). The protein structure of TgMAPK was obtained from the homology study. The docking procedure utilized a GRID of 10 Å radius and 0.30 resolution to cover the ligand-binding site in both PDB files. Root mean square distance (RMSD) was used to measure the average distance between the crystallized ligand and the ligand subjected to molecular docking, with an RMSD of up to 2.0 Å considered valid for docking. The Moldock scoring algorithm, along with the Moldock search algorithm, was employed according MVD user manual. MVD generated 5 poses for each molecule within the protein's active site. The most stable pose, characterized by the lowest interaction energy, was selected, and imported into Discovery Studio 2021 for visual inspection. The enzyme and compound structures were prepared using the default parameter settings in the software package, including ligand evaluation, number of runs, algorithm, maximum interactions, maximum population size, maximum steps, neighbour distance factor and maximum number of poses returned. Also, a decoy analysis was integrated to evaluate the reliability of the molecular docking approach. For this purpose, 50 decoy compounds specific to RWJ-67657 were included. The decoys were created using the DUD-E web tool (https://dude.docking.org/generate), specifically designed to generate suitable decoys for active compounds, as previously described (Mysinger *et al*., 2012).

### In vivo efficacy on mice infected by *T. gondii*

Mice were intraperitoneally inoculated with 10 cysts of the ME49 strain of *T. gondii* (Wang and Sibley, 2020). After 40 days of infection, the mice were randomly divided into experimental groups (*n* = 4 per group). These groups received 10 doses of RMJ-67657 (20 mg kg day^−1^, prepared with 4% DMSO in water) or PYR (20 mg kg day, prepared with distilled water) *via* oral gavage, with a final volume of 100 μL. The control group received 100 μL of the vehicle used to dilute RWJ-67657 (4% DMSO in water). The dose of 20 mg kg day^−1^ of RWJ-67657 was chosen based on previous work (Wei *et al*., 2007). Likewise, the decision to use the same dose of 20 mg kg day^−1^ of PYR was made to facilitate a direct comparison of efficacy between the 2 compounds. Throughout the treatment, signs of toxicity were assessed, considering parameters such as changes in body weight and behavioural alterations. On day 51 post-infection, all animals were euthanized, and their brains were collected for RNA extraction and quantification (Dos Santos *et al*., 2023). DNA extraction and purification were performed using the PureLink Genomic DNA Minikit (Invitrogen) following the manufacturer's instructions. For RNA extraction and purification from the brain tissue of experimental animals, the RNeasy Lipid Tissue Mini Kit (Qiagen) was used according to the manufacturer's instructions. After extraction, RNA samples were immediately processed for cDNA synthesis. The following steps were followed: Mix 1 was prepared using 11 μL of extracted RNA, 1 μL of DNTP and 1 μL of random primers. Mix 2 was prepared with 2 μL of DTT and 4 μL of buffer (Tris–HCl, 250 mm, pH 8.3; KCl, 375 mm; MgCl_2_, 15 mm). Moloney Murine Leukaemia Virus Reverse Transcriptase (M-MLV RT) was used for reverse transcription. Finally, 20 μL of cDNA was synthesized and stored at −20°C for future quantitative real-time (qPCR) analysis. The qPCR was performed on both DNA and cDNA samples using the Rep529 primer set, which amplified a repetitive DNA fragment of *T. gondii* (Pomares *et al*., 2020). The primer sequences were as follows: forward primer: 5′-GTTGGGAAGCGACGAGAGTC-3′, reverse primer: 5′-ATTCTCTCCGCCATCACCAC-3′, TaqMan probe FAM dye-labeled: 5′-AGAAGATGTTTCCGGCTTGGCTGCTT-3′ with NFQ as the quencher. Reactions were carried out using the Applied Biosystems™ StepOne™ Real-Time PCR System. Each reaction contained 3.5 μL of PCR water, 5 μL of 2X Universal TaqMan Master Mix, 0.25 μL of ‘primer mix’ (0.5 μL of both forward and reverse primers and 0.5 μL of the probe) and 1 μL of the sample. The thermal profile consisted of an initial step at 50°C for 2 min, followed by a denaturation step at 95°C for 10 min, and 40 cycles at 95°C for 15 s and 60°C for 1 min. Each run included a negative control (reaction mix without DNA) and a positive control (a highly concentrated sample previously obtained from culture). Prior to the experiment, reactions were standardized using templates with different concentrations of *T. gondii* DNA. A standard curve was used to correlate the cycle threshold value (C_T_) with varying amounts of parasites (Dos Santos *et al*., 2023).

### Statistical analysis

Statistical analysis was conducted to assess the significance of parasite burden in the brains of infected mice using One-Way ANOVA, followed by the Tukey post-test using the GraphPad Prism 8 software. Results were considered significant at a threshold of *P* < 0.05.

## Results

### Identification of highly active compounds against *T. gondii*

**Figure 1.**
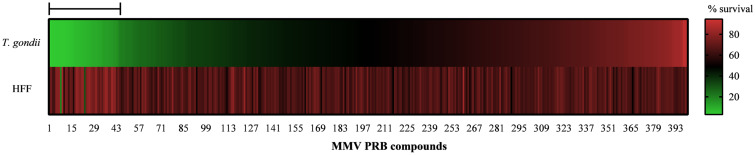
*In vitro* screening of PRB against *T. gondii* and HFF. A heat map depicting the per cent survival of *T. gondii* tachyzoites, as well as HFF cells when incubated with each of the 400 MMV PRB compounds at 1 μm, as described under ‘Experimental Procedures’. The horizontal bar indicates the 44 most active compounds.

A drug screening of 400 compounds provided by the MMV foundation in the PRB against *T. gondii* tachyzoites was performed using a *T. gondii* strain of the RH type I clone 2F1 encoding a transgenic copy of *β*-galactosidase. The screening aimed to select the most active compounds and was conducted at a single concentration of 1 μm. At 1 μm, 44 compounds were found to inhibit at least 80% of parasite viability, as depicted in Supplementary material. Additionally, the cytotoxicity of the same 400 compounds against HFF was screened at 1 μm to select the less cytotoxic compounds. Out of the 44 compounds that were able to inhibit more than 80% of parasite survival, only 2 compounds (MMV010036 – panobinostat and MMV1557856 – birinapant) inhibited more than 50% of HFF viability at 1 μm, as shown in Fig. 1.

### EC_50_ and CC_50_ determinations for selective compounds

A total of 42 compounds were selected, demonstrating the ability to inhibit over 80% of parasites' viability while having less than 50% impact on HFF viability. These compounds were further subjected to EC_50_ and CC_50_ determinations. This selection process enabled the identification of compounds with submicromolar anti-*T. gondii* activity and low cytotoxicity towards the host cell. Consequently, the analysis focused on these selective compounds. The chemical structures of these 42 selective compounds can be seen in Supplementary Material. The determined EC_50_ values for these compounds ranged from 2.4 to 913.1 nm, while the CC_50_ values ranged from 6.54 μm to >50 μm (the highest tested concentration was 50 μm). Seven compounds, namely itraconazole, trimetrexate, erythromycin, SQ109, clindamycin, iclaprim and RWJ-67657, had previously been described as anti-*T. gondii* agents. The SI values for each compound, calculated as the ratio between the CC_50_ and the EC_50_, varied from 11 to 17 708 (seventeen thousand seven hundred and eight). Notably, the compounds MMV1580844 and MMV1580173 (trimetrexate) exhibited the highest selectivity, as shown in Table 1.

**Table 1.** Comparative analysis of compounds *in vitro* activity

MMV code	Trivial name	*T. gondii*^a^	EC_50_ ± s.d. (nm)^b^	CC_50_ ± s.d. (μm)^c^	SI^d^	Main activity^e^
MMV1634492	Eberconazole	No	517.7 ± 243	46.29 ± 4	89.4	Antifungal (*n* = 7)
MMV002731	Ciclopirox	No	389.6 ± 130	>50	>128.3	
MMV637528	Itraconazole	Yes	206.7 ± 78	>50	>242.0	
MMV1634491		No	702.1 ± 273	29.26 ± 4	41.7	
MMV396785	Alexidine	No	583.4 ± 174	6.54 ± 3	11.2	
MMV1578560	OSU-03012	No	709.0 ± 228	19.73 ± 9	27.8	
MMV1782354	Olorofim	No	333.5 ± 216	>50	>149.9	
MMV1582497		No	578.9 ± 52	22.47 ± 2	38.8	Antibacterial (*n* = 25)
MMV1580173	Trimetrexate	Yes	4.7 ± 0.8	>50	>10,638.3	
MMV1633967		No	754.9 ± 324	>50	>66.2	
MMV003137	Erythromycin	Yes	695.8 ± 140	>50	>71.9	
MMV1581551		No	593.0 ± 76	>50	>84.3	
MMV021759		No	804.4 ± 254	49.19 ± 0.5	61.2	
MMV687800	Clofazimine	No	366.1 ± 93	>50	>136.6	
MMV1580840		No	842.2 ± 116	18.41 ± 8	21.9	
MMV1578842		No	554.4 ± 249	>50	>90.2	
MMV687273	SQ109	Yes	602.4 ± 89	48.95 ± 0.5	81.3	
MMV1593541		No	425.8 ± 82	26.13 ± 2	61.4	
MMV1581554	NSC 731130	No	668.5 ± 142	>50	>74.8	
MMV1580844		No	2.4 ± 0.5	42.50 ± 10	17 708.3	
MMV1634391		No	591.4 ± 73	>50	>84.5	
MMV1633674	Retapamulin	No	619.6 ± 35	>50	>80.7	
MMV689758	Bedaquiline	No	685.6 ± 239	>50	>72.9	
MMV011565	GNF-Pf-117	No	169.9 ± 65	>50	>294.3	
MMV000051	Clindamycin	No	751.3 ± 156	>50	>66.6	
MMV1634399		Yes	619.3 ± 208	29.75 ± 8	48.0	
MMV1581558		No	278.6 ± 47	>50	>179.5	
MMV1581549		No	135.2 ± 12	>50	>369.8	
MMV1578577	GW611920X	No	673.7 ± 211	>50	>74.2	
MMV233495	8-p-Tosylaminoquinoline	No	913.1 ± 94	>50	>54.8	
MMV1613563	Iclaprim	Yes	711.0 ± 243	>50	>70.3	
MMV1580843		No	569.3 ± 201	>50	>87.8	
MMV394033		No	686.8 ± 235	40.51 ± 12	59.0	Antiviral (n = 10)
MMV642550		No	291.9 ± 62	32.15 ± 17	110.1	
MMV1580794	Emricasan	No	557.3 ± 6	6.64 ± 4	11.9	
MMV1580797	RWJ-67657	Yes	479.1 ± 137	>50	>104.4	
MMV1782115		No	247.6 ± 62	9.30 ± 3	37.6	
MMV098836	DNDI1417411	No	484.3 ± 4	>50	>103.2	
MMV1580493	Verdinexor	No	438.1 ± 70	>50	>114.1	
MMV1580482	URMC-099-C	No	549.3 ± 246	26.13 ± 1	47.6	
MMV019724		No	204.6 ± 68	16.40 ± 11	80.2	
MMV1782211	TTP 8307	No	438.4 ± 117	>50	>114.1	
PYR	Pyrimetamine	Yes	299.9 ± 22	>50	>166.7	Anti-*T. gondii*

Activity against *T. gondii* tachyzoites, HFF cytotoxicity, and Selectivity Indexes (SI) of PRB selected compounds, with pyrimethamine (PYR) as reference drug.

a Known anti-*T. gondii* activity.

b Half effective concentration (EC_50_) against *T. gondii* tachyzoites.

c Half cytotoxic concentration (CC_50_) against HFF cells.

d Selectivity indexes (SI), calculated based on the CC_50_ HFF cells/EC_50_ *T. gondii* ratio.

e Known main biological activity.

### Predicted ADMET properties and candidate selection

**Figure 2.**
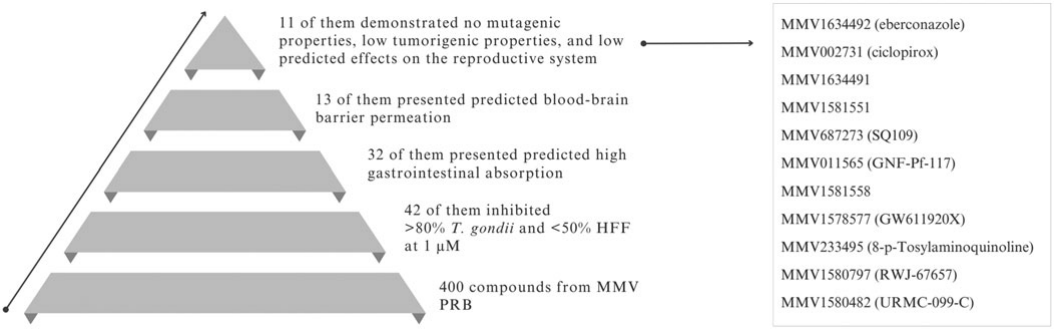
Summary of screening results and identification of top 11 promising new compounds for experimental chronic toxoplasmosis. This figure provides an overview of the screening results obtained from 400 compounds of the PRB and highlights the selection of the 11 most promising anti-*T. gondii* agents for further investigation in experimental model of chronic cerebral toxoplasmosis. The compounds were evaluated based on their *in vitro* selective activity against *T. gondii* and their ADMET proprieties.

Out of the 42 selected compounds, 32 demonstrated high gastrointestinal absorption, and 13 exhibited blood–brain barrier permeation (Table 2). Among these, 11 compounds also demonstrated no mutagenic properties, low tumorigenic properties, and low predicted effects on the reproductive system and were considered promising candidates for experimental cerebral toxoplasmosis (Fig. 2). Based on desirable ADMET predictions and commercial availability, compound MMV1580797 (RWJ-67657) was selected for further investigation.

**Table 2.** *In silico* predictions of pharmacokinetics, drug likeness and toxicity for selected compounds

MMV code	ID	GI absorption	BBB permeation	Lipinski filter^a^	Toxicity		
					Mutagenic	Tumorigenic	Reproductive effect
MMV1634492	1	High	Yes	1	None	None	None
MMV002731	2	High	Yes	0	None	None	None
MMV637528	3	High	No	2	High	High	None
MMV1634491	4	High	Yes	0	None	None	None
MMV396785	5	Low	No	2	None	None	High
MMV1578560	6	Low	No	0	High	High	None
MMV1782354	7	High	No	0	High	High	None
MMV1582497	8	High	No	0	None	None	None
MMV1580173	9	High	No	0	None	None	None
MMV1633967	10	Low	No	1	None	None	None
MMV003137	11	Low	No	2	None	None	None
MMV1581551	12	High	Yes	0	None	None	None
MMV021759	13	High	No	0	None	None	None
MMV687800	14	Low	No	1	None	None	None
MMV1580840	15	Low	No	2	Low	High	High
MMV1578842	16	High	No	0	None	None	None
MMV687273	17	High	Yes	1	None	None	None
MMV1593541	18	Low	No	0	High	None	None
MMV1581554	19	High	No	0	None	None	None
MMV1580844	20	High	No	0	High	Low	High
MMV1634391	21	High	No	2	None	None	None
MMV1633674	22	High	No	1	None	None	None
MMV689758	23	Low	No	2	Low	High	None
MMV011565	24	High	Yes	0	None	None	None
MMV000051	25	High	No	0	High	High	None
MMV1634399	26	High	Yes	1	High	None	None
MMV1581558	27	High	Yes	0	None	None	None
MMV1581549	28	High	No	0	High	Low	High
MMV1578577	29	High	Yes	0	None	None	None
MMV233495	30	High	Yes	0	None	None	None
MMV1613563	31	High	No	0	None	None	None
MMV1580843	32	High	No	1	None	None	None
MMV394033	33	High	Yes	0	High	High	High
MMV642550	34	High	No	0	None	High	None
MMV1580794	35	Low	No	1	None	None	None
MMV1580797	36	High	Yes	0	None	None	None
MMV1782115	37	High	No	0	None	None	None
MMV098836	38	High	No	0	High	None	None
MMV1580493	39	High	No	0	None	None	Low
MMV1580482	40	High	Yes	0	None	None	None
MMV019724	41	Low	No	0	None	High	None
MMV1782211	42	High	No	0	None	None	None
PYR	43	High	Yes	0	High	High	High

Predictions of gastrointestinal (GI) absorption, blood–brain barrier (BBB) permeation, Lipinski filter compliance and toxicity (mutagenicity, tumorigenicity and reproductive effects) of PRB selected compounds compared to pyrimethamine (PYR) as the reference drug.

a Lipinski (Pfizer) filter: molecular weigh ⩽ 500; MLogP ⩽ 4.15; N or O ⩽ 10; NH or OH ⩽ 5.

### Molecular docking analysis of TgMAPK1 interaction

**Figure 3.**
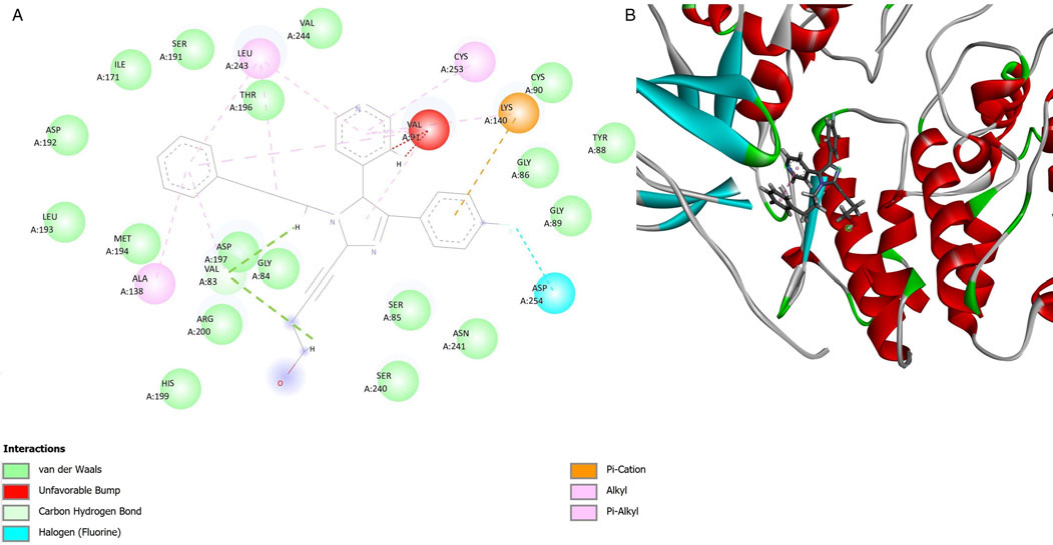
Molecular docking analysis of *T. gondii* p38 mitogen-activated protein kinase (TgMAPK1) with compound RWJ-67657. The docking analysis demonstrated a high probability of compound RWJ-67657 interacting with the active site, forming significant interactions with several amino acid residues. 2D interaction representation (A) and 3D interaction representation (B).

Molecular docking analysis of *T. gondii* p38 mitogen-activated protein kinase (TgMAPK1) with compound RWJ-67657 revealed that it is predicted to bind to this protein (Fig. 3). A hybrid model of TgMAPK1 was generated based on a previous study (Brumlik *et al*., 2004), and the Ramachandran plot showed that over 97.2% of residues were in the most favoured regions, with 99.7% of residues in allowed regions. The active site region of the TgMAPK1 protein contains amino acid residues including Ser191, Asp192, Met194, Asp197, Ser240, Val91, Lys238, Asn241, Ala87, Gly89, Gly256, Glu158, Tyr88, Lys140 and Asp254. In the docking analysis, it was observed that the compound RWJ-67657 exhibited a high probability of interacting with this active site, forming significant interactions with several of these amino acid residues. These interactions included van der Waals interactions with Ser191, Asp192, Met194, Asp197, Ser240, Asn241, Gly89 and Tyr88 residues, halogen (fluorine) interaction with the Asp254 residue, and Pi-Cation interaction with residue Lys140. The compound demonstrated an interaction energy probability of −152.65 kcal mol^−1^. The compound pyrimethamine was used as a positive control in the docking analysis, and it exhibited an interaction energy probability of −86.416 kcal mol^−1^. Therefore, the compound RWJ-67657 showed a greater stability of interaction in the active site of the TgMAPK1 protein.

An analysis with decoys was also conducted to assess the performance and robustness of the molecular docking study. These decoys were created using DUD-E, a web tool designed to generate decoys for active compounds. In this instance, decoys were generated for the compound RWJ-67657. A set of 50 compounds was generated and subsequently used in the molecular docking analysis. Remarkably, among these 50 decoy compounds, only 1 exhibited lower energy values compared to RWJ-67657. This outcome significantly reinforces the robustness and reliability of the docking study.

### Selectivity assessment against human p38 MAPK

To evaluate the selectivity of compound RWJ-67657 for the TgMAPK-1 protein, a molecular docking analysis was performed using the human p38 MAPK protein (PDB ID: 6Y4T) as a reference (Röhm *et al*., 2020). As a positive control, the inhibitor SR63, previously crystallized with the protein, was employed. The analysis revealed an interaction energy of −216.59 kcal mol^−1^ for the inhibitor SR63 and −123.78 kcal mol^−1^ for compound RWJ-67657. This disparity suggests a low likelihood of interaction with the human p38 MAPK. Consequently, compound RWJ-67657 demonstrates a higher potential for selectivity toward the TgMAPK-1 protein compared to p38 MAPK.

### Molecular dynamics analysis for interaction stability

**Figure 4.**
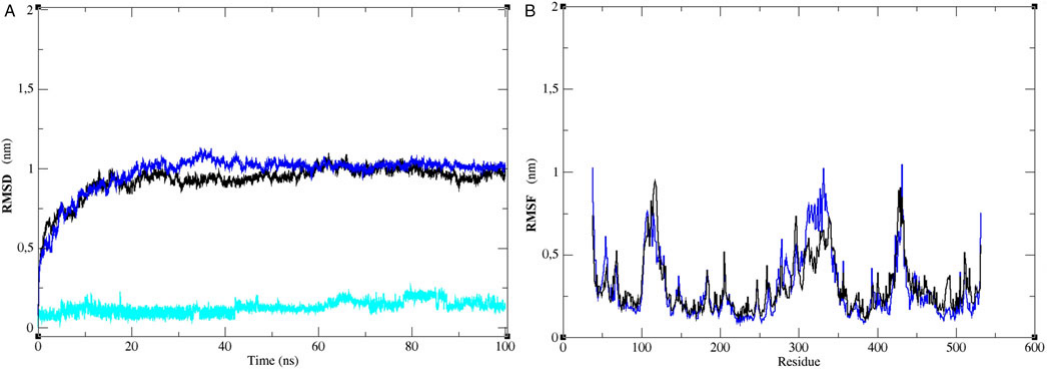
Molecular dynamics analysis of protein–ligand interaction stability. Root mean square deviation (RMSD) (A) and Root mean square fluctuation (RMSF) (B) within the TgMAPK binding site. The black line indicates the TgMAPK, the blue line indicates the TgMAPK:RWJ-67657 complex and the cyan line indicates the ligand RWJ-67657.

To further assess the stability of TgMAPK1 interaction with compound RWJ-67657, a molecular dynamics analysis was conducted. Initially, root mean square deviation (RMSD) analyses were performed to evaluate the structural stability of the receptor framework. These analyses measured the distance between different positions of a set of atoms over time (in nm). Similar levels of perturbation were observed during the first 20 ns, with RMSD values ranging from 0.10 to 1 nm for both the protein alone and the protein-RWJ-67657 complex. After 30 ns, the protein in the complex exhibited similar stability to the protein alone. The structure of RWJ-67657 consistently demonstrated stability within the active site of the protein, with a variation of 0.1–0.2 nm throughout the 100 ns simulation (Fig. 4A).

Next, the residue flexibility was analysed using root-mean-square fluctuation (RMSF) values for the protein–ligand complex (Fig. 4B). Similar patterns were observed between the protein alone and the protein–ligand complex throughout the entire simulation, indicating the stability of the interaction between the molecule and the protein, with no significant variations in the protein that could impair the interaction.

### In vitro assessment of RWJ-67657 against *T. gondii* tachyzoites

**Figure 5.**
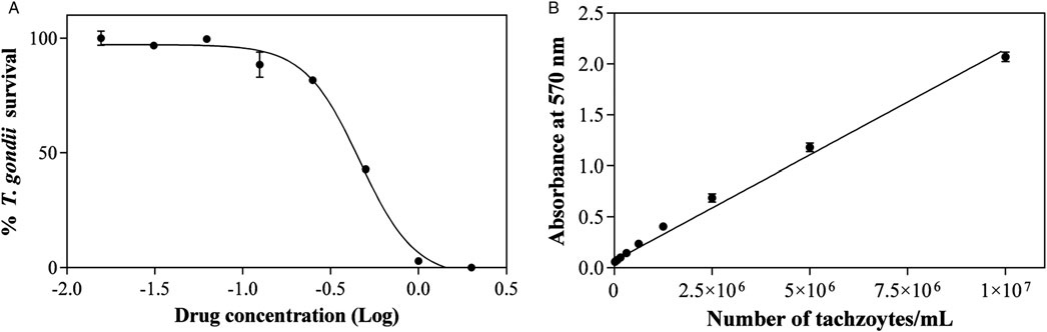
Effect of RWJ-67657 treatment on intracellular *T. gondii* tachyzoites. Infected HFF cells were treated with RWJ-67657 at different concentrations. The dose–response curve of RWJ-67657 was determined by quantifying the activity of *β*-galactosidase, with the x-axis representing the logarithm of the drug concentration in micromolar (A). Correlation between absorbance values and the number of 2F1 *T. gondii* tachyzoites per mL using the CPRG substrate for *β*-galactosidase quantification (B).

The dose–response curve of RWJ-67657 on intracellular *T. gondii* tachyzoites determined by *β*-galactosidase quantification is shown in Fig. 5A. The correlation between absorbance values and the number of 2F1 *T. gondii* tachyzoites per mL using the CPRG substrate for *β*-galactosidase quantification was confirmed through the construction of a standard curve ranging from 10^7^ to 2 parasites. A linear correlation between *β*-galactosidase activity and the number of parasites was found (*r*^2^ = 0.99) (Fig. 5B). Moreover, this assay could discern a parasite concentration as low as 2 tachyzoites per mL.

### In vivo efficacy of RWJ-67657 in *T. gondii*-chronically infected mice

**Figure 6.**
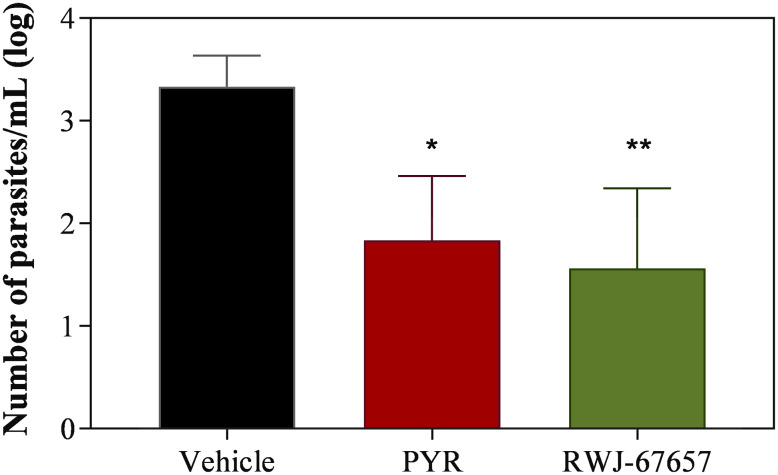
*In vivo* assessment of compound RWJ-67657 efficacy against chronic *T. gondii* infection in mice. Grouped column graph showing the impact of oral gavage administration of 20 mg kg day^−1^ RWJ-67657 or pyrimethamine (PYR) over 10 consecutive days on the number of live parasites in the brains of C57BL/6 mice infected with *T. gondii* (ME49 strain), in comparison to vehicle-treated mice, estimated by RT-qPCR. The asterisk denotes a statistically significant difference of pyrimethamine (*P* < 0.05) and RWJ-67657 (*P* < 0.01) compared to the vehicle-treated group.

C57BL/6 mice were infected with the ME49 strain of *T. gondii* and 40 days post-infection were orally administered with RWJ-67657 at a dose of 20 mg kg day^−1^ for 10 consecutive days. A group of animals treated with the standard drug pyrimethamine at the same dose served as a positive control, while a group that received the mock vehicle served as a negative control. The RT-qPCR quantification of parasites in the brain of infected animals indicated a statistically significant reduction in parasite burden in the group treated with RWJ-67657 (*P* < 0.01) and with the clinically used drug pyrimethamine (*P* < 0.05) when compared to the vehicle-treated group (Fig. 6). No signs of toxicity, including changes in body weight and behavioural alterations, were observed during drug treatment.

## Discussion

In the pursuit of new drug candidates for toxoplasmosis, a promising strategy is the drug repurposing, which involves investigating existing drugs for new therapeutic applications. This approach offers several advantages, including shorter development timelines, reduced costs and the potential to uncover novel therapies from known compounds (Da Silva *et al*., 2021). In this regard, the MMV boxes have emerged as a valuable resource of anti-*T. gondii* compounds with known pharmacological properties. The MMV boxes offer a valuable starting point in the quest for new therapies against toxoplasmosis, providing a platform for drug repurposing and enabling the discovery of innovative treatment options (Spalenka *et al*., 2018; Da Silva *et al*., 2021; Dos Santos *et al*., 2023).

Screening of the MMV PRB resulted in the identification of 42 compounds that exhibited selective anti-*T. gondii* activity. These compounds encompassed 7 antifungals, 25 antibacterials and 10 antivirals. Importantly, the screening process led to the discovery of new anti-*T. gondii* drug candidates, along with compounds that served as satisfactory external controls due to their known anti-parasitic properties. In addition, PYR was utilized as a reference drug, and the obtained EC_50_ value aligned with previously reported values for the inhibitory activity of this DHFR inhibitor on *T. gondii* growth *in vitro* (Radke *et al*., 2018).

Among the compounds tested, the most potent inhibitors of *T. gondii* were MMV1580173 (trimetrexate) and MMV1580844, both demonstrating EC_50_ values in the nanomolar range and exhibiting the highest SI values. Trimetrexate, previously documented to possess *in vitro* and *in vivo* anti-*T. gondii* activity, functions as an inhibitor of *T. gondii* DHFR (Kovacs *et al*., 1987). Notably, this study presents the first description of the anti-*T. gondii* activity of MMV1580844 (CHEMBL2335419). Previous research has identified it as an anti-*Staphylococcus aureus* agent targeting DHFR (Frey *et al*., 2012), and it is likely that it exerts a similar DHFR-targeting effect in *Toxoplasma*. Furthermore, the compound adheres to Lipinski's rule of 5, indicating its potential as a candidate for further investigation in *T. gondii*-infected mice.

Among the 42 identified anti-*T. gondii* compounds, RWJ-67657 was selected for further evaluation. This compound was chosen based on several factors: its nanomolar *in vitro* activity against *T. gondii*, a SI greater than 100, no previous reports on its *in vivo* efficacy against chronic toxoplasmosis, predicted high gastrointestinal absorption, predicted ability to cross the blood–brain barrier (essential for targeting bradyzoites within brain cysts), commercial availability and predicted absence of mutagenic properties, low tumorigenic properties and low predicted effects on the reproductive system.

RWJ-67657 is a p38 MAPK inhibitor (Westra *et al*., 2004). MAPKs govern diverse cellular processes including proliferation and differentiation. A previous work showed that treatment of *T. gondii*-infected cells with substituted pyridinylimidazoles that are potent inhibitors of human p38 MAPK, inhibits intracellular *T. gondii* replication. The authors suggest that the anti-proliferative effects of pyridinylimidazoles depend on direct action on tachyzoites, not the host cell through several approaches. They hypothesize that pyridinylimidazoles target a human p38 MAPK homologue in tachyzoites that regulates their replication (Wei *et al*., 2002). Phylogenetic data indicate that *T. gondii* possesses a p38 MAPK homologue called TgMAPK-1. This protein has been identified as a novel drug target for anti-*T. gondii* therapy due to its unique characteristics, including 3 previously unreported amino acid insertion sequences (Brumlik *et al*., 2004).

The presented molecular docking results suggested that compound RWJ-67657 is a potent inhibitor of TgMAPK1. This finding proposes that the observed anti-proliferative effect of compound RWJ-67657 can be attributed to its interaction with this protein and reinforces the potential of p38 MAPK inhibitors as anti-*T. gondii* agents (Wei *et al*., 2002).

RWJ-67657 exhibited an anti-proliferative effect on *T. gondii* tachyzoites, as evidenced by *β*-galactosidase quantification. The inhibitory effect of RWJ-67657 on intracellular tachyzoites demonstrated a dose-dependent response, while maintaining the integrity of the host cells at the tested concentrations. Moreover, administering compound RWJ-67657 to mice at a dose of 20 mg kg day^−1^ for 10 consecutive days resulted in a significant 96.3% reduction in parasite burden within the brain. This finding suggests that the drug effectively crosses the blood–brain barrier and achieve therapeutic concentrations within brain tissue. Furthermore, it indicates that extending the treatment duration or increasing the drug dosage may potentially yield even greater therapeutic effects.

Previous studies have shown the *in vivo* efficacy of RWJ-67657 in treating acute *T. gondii* infection in CBA/J mice infected with the RH strain, as evidenced by improved survival rates (Wei *et al*., 2007). However, this is the first report investigating the effect of this compound in the treatment of chronic toxoplasmosis in C57BL/6 mice infected with the ME49 strain of *T. gondii.* In this study, drug efficacy was verified by quantifying the parasite burden using RT-qPCR. Although there are drugs available for controlling acute toxoplasmosis, an effective therapy for treating chronic toxoplasmosis is currently lacking. The bradyzoites, which reside within *T. gondii* cysts commonly found in the brain, eyes and muscles, demonstrate resistance to existing drugs. This resistance highlights the urgent need for the development of well-tolerated treatments capable of eliminating this persistent stage of the parasite. Given the growing population of high-risk individuals, especially immunocompromised patients, sustained research efforts are crucial in identifying novel drug candidates to effectively combat chronic toxoplasmosis (Montazeri *et al*., 2018).

Although there are medications available for the treatment of toxoplasmosis, they may not always be suitable due to adverse reactions or therapeutic failures related to toxicity and parasite resistance to the drugs. For example, pyrimethamine cannot be used before 14 weeks of gestation for the potential risk of teratogenicity (Bollani *et al*., 2022). This risk has been highlighted also *in silico*, indicating that pyrimethamine carries toxicity alerts, including potential risks of tumorigenicity, mutagenicity and reproductive effects. In contrast, *in silico* predictions do not indicate any toxicity risks associated with the compound RWJ-67657. A previous study has shown that single oral doses of RWJ-67657 (from 0.25 to 30 mg kg^−1^) did not result in significant adverse effects, suggesting acceptable safety and pharmacokinetics for further investigation in a repeat-dose setting (Parasrampuria *et al*., 2003). In the present study, daily doses of 20 mg kg^−1^ were administered to mice. Converting this dose from animals to humans, using the Body Surface Area (BSA) as a factor, as proposed by Reagan-Shaw *et al*. (2008), the translation human dose is 1.62 mg kg day^−1^. Therefore, it is highly likely that compound RWJ-67657 would achieve effective doses in humans and that this dose would be well-tolerated. However, additional studies are required to confirm this hypothesis.

Corroborating the *in vitro* findings, the efficacy of RWJ-67657 extends beyond its protective effects against acute toxoplasmosis in mice, as it also exhibits the ability to reduce the parasite burden in chronically infected animals. These results support the potential repurposing of RWJ-67657 as an effective candidate against acute and chronic toxoplasmosis.

Moreover, 11 compounds with desirable ADMET properties and selective anti-*T. gondii* activity were identified, thereby being considered promising candidates for evaluation against experimental chronic cerebral toxoplasmosis. Consequently, this study opens new insights for future research aimed at discovering new options for toxoplasmosis chemotherapy.

The results presented in this study demonstrate that the MMV PRB is a promising source of anti-*T. gondii* compounds, with many displaying favourable physicochemical drug properties and thus representing good candidates for further investigation in experimental models of *T. gondii* infection. Furthermore, the findings suggest that RWJ-67657 has the potential to be a valuable drug candidate for the development of experimental therapies for toxoplasmosis, as it was able to significantly reduce parasite burden in the brain tissue of ME49 infected mice. It is important to emphasize that the absence of an effective treatment for chronic toxoplasmosis highlights the significance of discovering a new compound with the ability to eliminate the parasite within cerebral cysts. This discovery holds significant relevance in the field of drug discovery for toxoplasmosis.

## Supporting information

dos Santos et al. supplementary material 1 dos Santos et al. supplementary material

dos Santos et al. supplementary material 2 dos Santos et al. supplementary material

## Data Availability

The authors confirm that all the data supporting the findings presented in the present study are available in the manuscript. Raw data are available from the corresponding and last author.

## Supplementary material

The supplementary material for this article can be found at https://doi.org/10.1017/S0031182023000999
