# Supplementary material for: Anti-Toxoplasma gondii screening of MMV pandemic response box and evaluation of RWJ-67657 efficacy in chronically infected mice
Source: Parasitology. 2023 Oct 20;150(13):1226–35. doi: 10.1017/S0031182023000999 (PMC10941209; doi:10.1017/S0031182023000999)
Supplement: dos Santos et al. supplementary material 2 — dos Santos et al. supplementary material [file S0031182023000999sup002.pdf]

Supplementary material. Chemical structures of 42 anti-*T. gondii* selective compounds from MMV Pandemic Response Box.

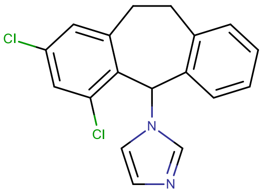

MMV1634492
Eberconazole

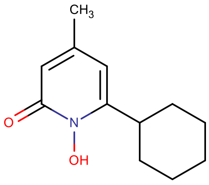

MMV002731
Ciclopirox

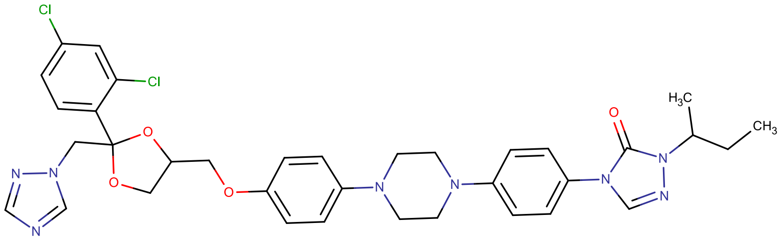

MMV637528
Itraconazole

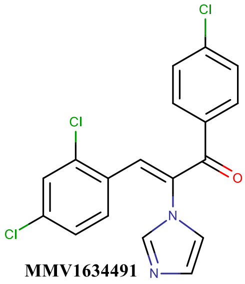

MMV1634491

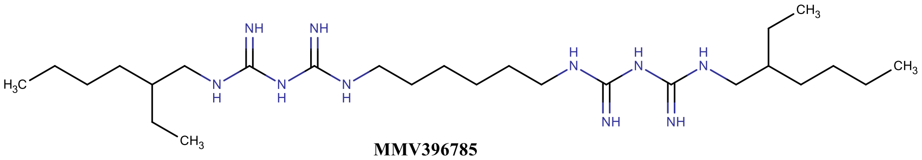

MMV396785
Alexidine

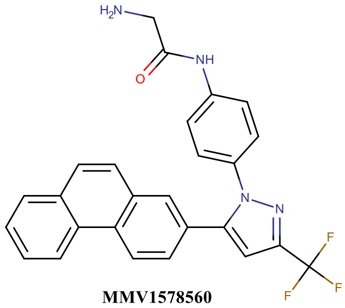

MMV1578560
OSU-03012

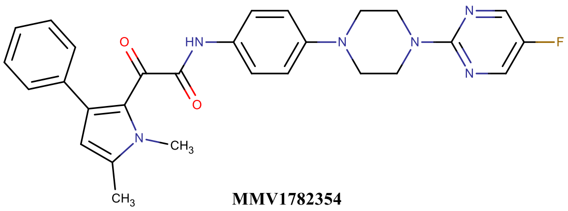

MMV1782354
Olorofim

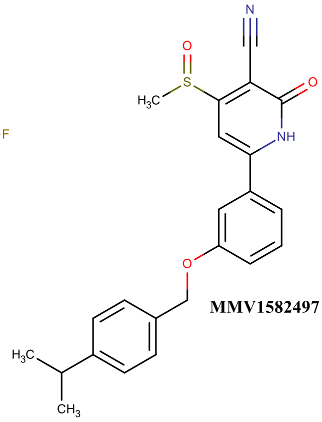

MMV1582497

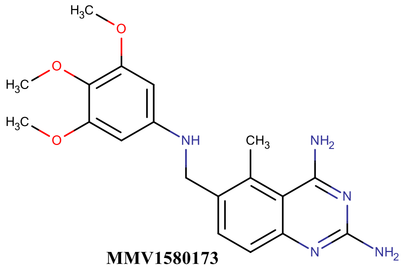

MMV1580173
Trimetrexate

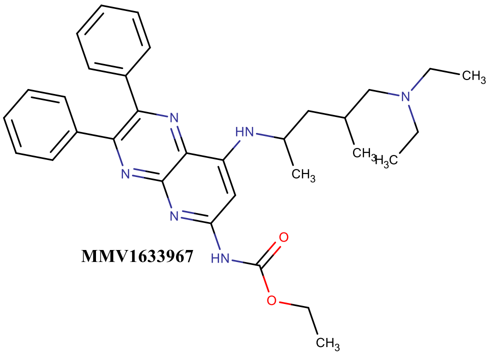

MMV1633967

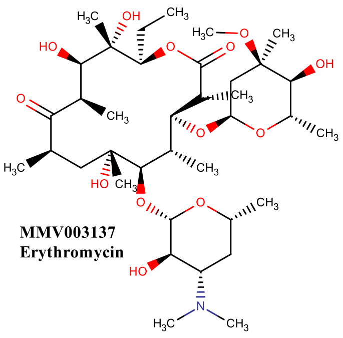

MMV003137
Erythromycin

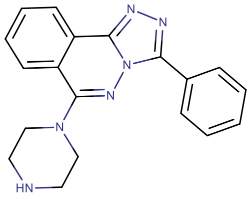

MMV1581551

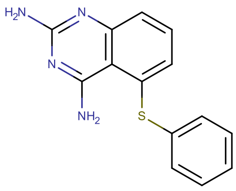

MMV021759

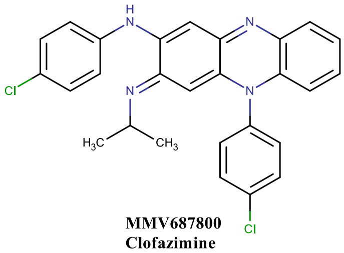

MMV687800
Clofazimine

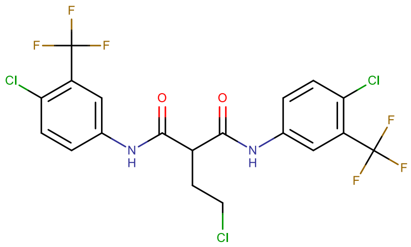

MMV1580840

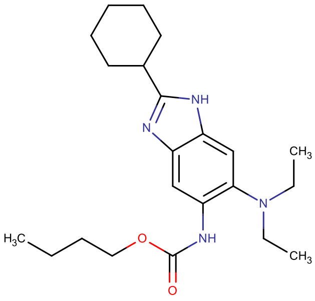

MMV1578842

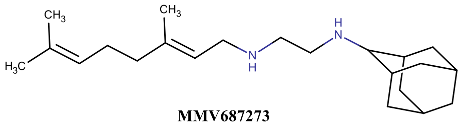

MMV687273
SQ109

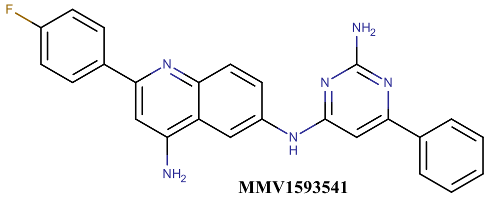

MMV1593541

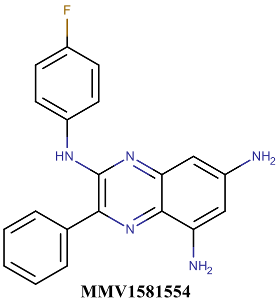

MMV1581554
NSC 731130

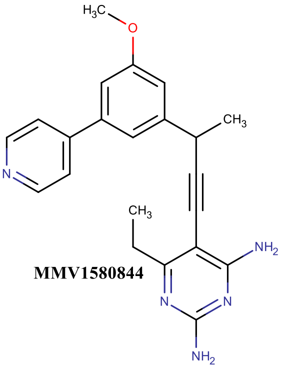

MMV1580844

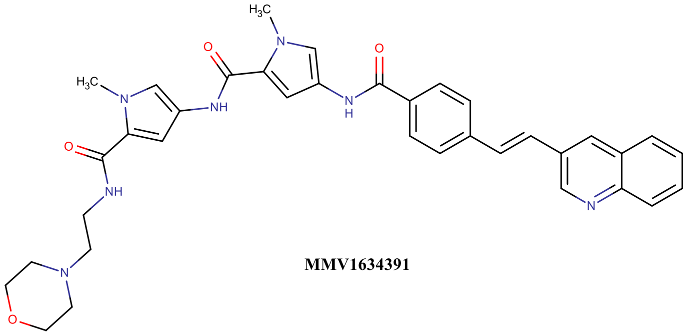

MMV1634391

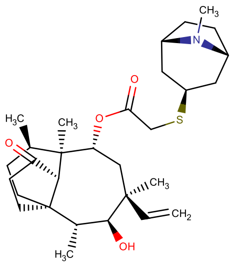

MMV1633674
Retapamulin

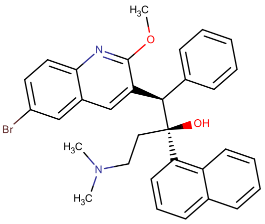

MMV689758
Bedaquiline

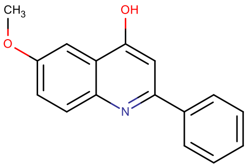

MMV011565
GNF-Pf-117

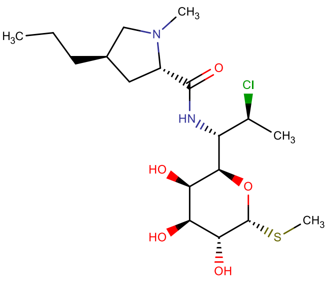

MMV000051
Clindamycin

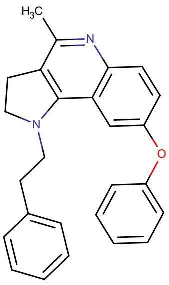

MMV1634399

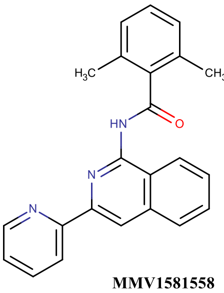

MMV1581558

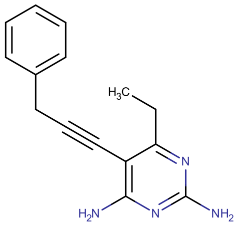

MMV1581549

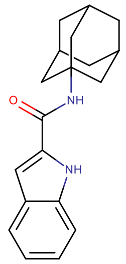

MMV1578577
8-p-Tosylaminoquinoline

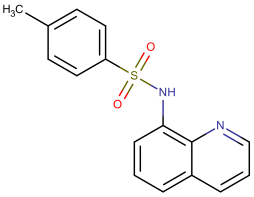

MMV233495

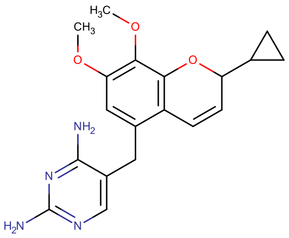

MMV1613563
Iclaprim

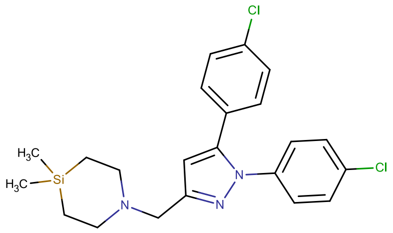

MMV1580843

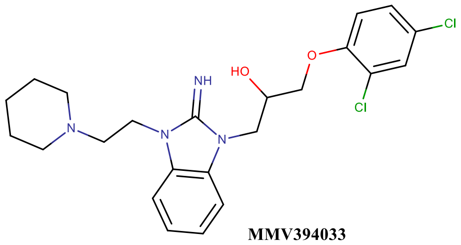

MMV394033

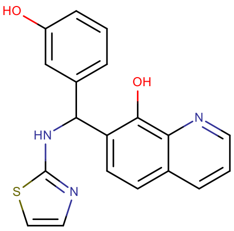

MMV642550

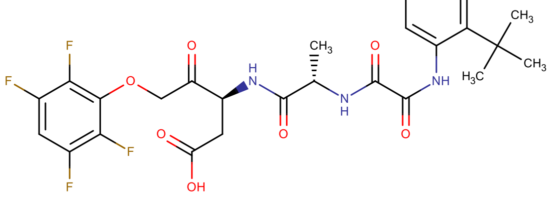

MMV1580794
Emricasan

MMV1580797
RWJ-67657

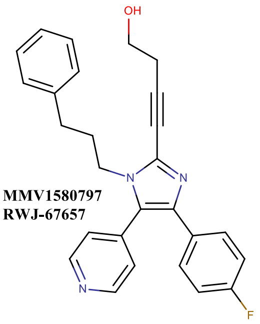

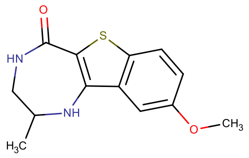

MMV1782115

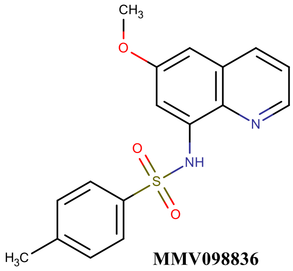

MMV098836
DNDI1417411

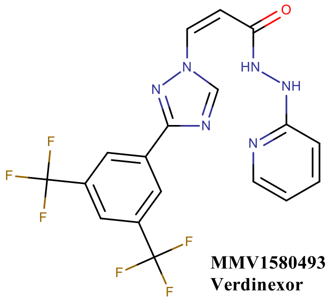

MMV1580493
Verdinexor

MMV1580482
URMC-099-C

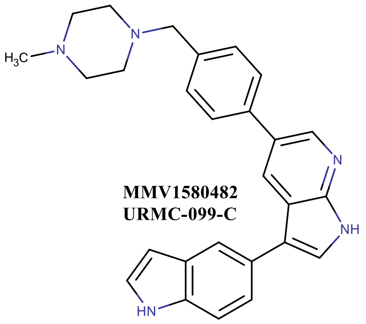

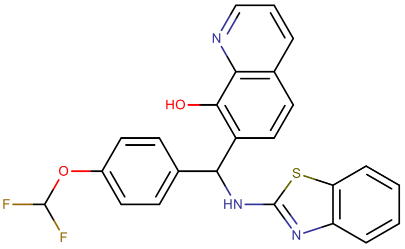

MMV019724

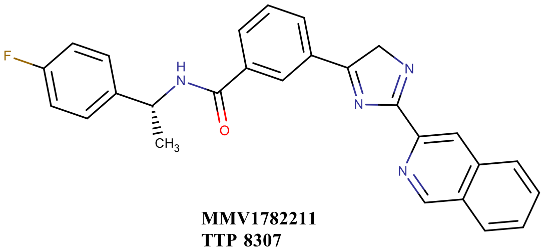

MMV1782211
TTP 8307
